# OSVALDO MALAFAIA, FORMER PRESIDENT OF THE BRAZILIAN COLLEGE OF DIGESTIVE SURGERY

**DOI:** 10.1590/0102-6720202400037e1831

**Published:** 2025-02-28

**Authors:** Nicolau Gregori CZECZKO, Jurandir Marcondes RIBAS

**Affiliations:** 1 Universidade Federal do Paraná, Department of Surgery - Curitiba (PR), Brazil; 2 Mackenzie Evangelical College of Paraná, Department of Surgery - Curitiba (PR), Brazil

To frame this editorial on Osvaldo Malafaia, we ask our readers to bear with our poetic license so that we can freely set about creating a flexible framework about his life pathway. These are conjectures regarding what may have happened, as well as facts that did magnificently take place.

The year is 1963. Osvaldo, 17 years old, from the town of Campinas, has begun to read medicine at the Federal University of Paraná in Curitiba. His accommodation is at the Medical and Surgical Sanatorium in the rather distant borough of Portão, an exclusive mandatory health center for TB patients at the time, and an hour-and-a-half bus ride from the medical school classes. That commute enabled him to revise the daily knowledge content. At that sanatorium, he would attend procedures at the surgical center, following them up, and occasionally performing pulmonary decortications and phrenicotripsies, complications of pulmonary infection ensuing from Koch’s bacillus.

During one of his classes at the university anatomy amphitheatre, Osvaldo was meticulously undertaking a cadaver dissection of a forearm, specifically at the division of the radial nerve below the brachioradialis muscle and its superficial and deep branches. Suddenly, chatting is hushed, and not even any whispering is ventured, and sounds swiftly begin to hide themselves behind the silence. Professor Brasílio Vicente de Castro (1913-1978), Anatomy Chair, *magister dixit,* in his teaching and academic supervision walks in. One of the major anatomists in Brazil, a disciple of the classical school of Jean Léon Testut (1849-1925). His two assistants follow the Professor, respectfully standing behind him. He scans the register and walks between the benches. He stops at bench seven, with a gaze laden with some curiosity. He observes the diligent and elegant dissection, which had been presented in detail in his theoretical lesson, and asks,

- *Quo vadis,* Osvaldo?

Intelligent, organized, methodical, generous, altruistic, responsible, hard-working, companion, friend, explorer, and builder; in Osvaldo’s brain, complex cognitive processes are triggered as sparks in neuronal connections of the prefrontal and temporal cortex, as he devised his answer. With his agile and efficient thought, his plans, wishes, aspirations, and aims of a good man take shape. Aristotelian musings regarding self-knowledge and self-improvement in character building, reasoning, human function, excellence in life, happiness, and flourishing through virtue shine through. With the neurotransmitter release, the answers in the mind become clear:

*I want to become a doctor (1963-1968), to pursue a postgraduate degree (residence in surgery, 1969-1970, PhD,1974-1976), to be a lecturer (full professor in 1990 and Emeritus 2016, the Federal University of Paraná (UFPR), Full Professor at the Faculdade Evangélica Mackenzie do Paraná (FEMPAR) since 2001), to learn from other educational institutions, to get married (Sônia), to have three children (Marcelo, Maurício and Marilena), to have grandchildren (Nina, Paola, Marcela, Thiago and Matheus, the latter a student at FEMPAR in Curitiba); to speak at conferences (979 activities at events), to write journal articles (published 521), to supervise postgraduate students (126 MA and 67 PhD), to write books (6 books and 87 book chapters published), to help undergraduate and postgraduate students (336 undergraduate students at FEMPAR, and 470 at the UFPR, MA and PhD students over 39 years), to participate in degree exam boards (101), and to organise scientific events (109).*

*Nota Bene: Osvaldo Malafaia graduated from medicine at the age of 22, in the year that Dr. Euryclides de J. Zerbini (1912-1993) carried out the first heart transplant in Latin America (1968), he became a university lecturer in the same year that Dr. Baruch S. Blumberg (1925-2011) became the Nobel Prize winner for identifying the Hepatitis B virus (1976). With Prof. Henrique Walter Pinotti (1929-2010), and other major digestive surgery specialists, he was a founder member of the Brazilian College of Digestive Surgery in the year in which Single Health System of Brazil, SUS, was launched (1988)^3,4^. He chaired the Brazilian Gastroenterology Federation until the year of the fourth football championship which the Brazilian team conquered in the USA (1992-1994), and served as the editor-in-chief of the journal Arquivos Brasileiros de Cirurgia Digestiva (The Brazilian Archives of Digestive Surgery), for twenty-one years, starting in the year when Leland H. Hartwell, Tim Hunt and Sir Paul Nurse were awarded the Nobel Prize for their discoveries of key regulators of the cell cycle (2001)^1,2,5^.*

Only 2 or 3 s have gone after the initial question,

“Whither goest thou, Malafaia?”

The silence in the anatomy amphitheatre was thick, one could cut it with a scalpel. And Professor Brasílio Vicente de Castro, as if foreseeing future events with his intuitive precognition, rephrased his question turning it into advice,

- Malafaia, *Venire, Studium et Vincere*!

The advisory message prescribed a long journey. It was not about registering to read medicine, but it was about facing numerous challenges from graduation onwards. It meant studying always to learn, to teach, and to succeed in profession and life. Therefore, presently, on account of that grueling advice, Professor Osvaldo Malafaia stands among the most important medical professors/academics in Brazil; he is a virtuous winner, indeed!

Fifty-one years on, questions about Osvaldo Malafaia’s legacy have been fizzing brought about by curiosity and admiration to acclaim his achievements due to the absolute and exciting *status quo* in which we are close and often together on the pathway of a positive journey toward a solid scientific edifice and an uplifting symbiotic relation with goodness. Yes, he did create two *stricto sensu postgraduate* degree programmes (UFPR in 1988, and FEMPAR in 1992, Master’s and PhD), he served as the chair of the International College of Surgeons, USA (1996-1998) in Brazil and, at the turn of the century, served as the Chair of the Brazilian College of Digestive Surgery (1999-2000), and as the Medicine III area coordinator at the Foundation for the Coordination for the Improvement of Higher Education Staff (CAPES) of the Ministry of Education of Brazil (1997-2000), and as the deputy director and director of Health Sciences Section of the “UFPR” (1989-1990), he set up the “Semanas Brasileiras do Aparelho Digestivo” (Brazilian Digestive System Apparatus Study Weeks) (CBCD/FBG/SOBED, 1994), he is the editor “Revista BioSCIENCE” of the Paraná Medical Association, and Emeritus Member, patron of chair no. 56 of the Paraná Academy of Medicine; his citation rankings are thirteenth in Surgery and sixth in Digestive Surgery (2023). He was honored by having his name inscribed on the yearly CBCD Medal for the top article in the journal “Revista ABCD.”

The doctor, surgeon, professor, researcher, and leader represent the highest level of standing of those who are unique, irreplaceable, the best of the best, from where the brilliance of his life also enlightens us, and as a professional role model, he is truly inspirational.

There are exceptional seconds and minutes in life. Such was the rare idyllic spare time moment enjoyed last week. Sitting in the back garden at home, Malafaia was able to listen to the melodious song of the bird, which is the symbol of Brazil, the rufous-bellied thrush (*sabiá-laranjeira*). Nina, 7 years old, who had just come back from school, was cuddling her dolly. Out of the blue, fondly, she pointed her little finger and said, “Hi Grandpa!” Next, she started to clap her hands! Her clapping hands were joined in with those of thousands of patients, thousands of students, hundreds of trainee doctors, and of his friends. Similarly to the chirping of the *sabiá*, an orchestra of sounds impersonated the broadest appreciation for his life achievements!

And when we asked him, “Professor Doctor Osvaldo Malafaia, how do you view this pathway of yours?”

- “With joy,” he replied.

And what about the future?

- “With vehement enthusiasm, a bearer of good tidings!”
